# Ambulatory Healthcare Use Profiles of Patients With Diabetes and Their Association With Quality of Care: A Cross-Sectional Study

**DOI:** 10.3389/fendo.2022.841774

**Published:** 2022-04-13

**Authors:** Julien Dupraz, Emilie Zuercher, Patrick Taffé, Isabelle Peytremann-Bridevaux

**Affiliations:** Center for Primary Care and Public Health (Unisanté), University of Lausanne, Lausanne, Switzerland

**Keywords:** diabetes mellitus, ambulatory care, profiles, cluster analysis, quality of health care, process assessment, outcome assessment

## Abstract

**Background:**

Despite the growing burden of diabetes worldwide, evidence regarding the optimal models of care to improve the quality of diabetes care remains equivocal. This study aimed to identify profiles of patients with distinct ambulatory care use patterns and to examine the association of these profiles with the quality of diabetes care.

**Methods:**

We performed a cross-sectional study of the baseline data of 550 non-institutionalized adults included in a prospective, community-based, cohort study on diabetes care conducted in Switzerland. Clusters of participants with distinct patterns of ambulatory healthcare use were identified using discrete mixture models. To measure the quality of diabetes care, we used both processes of care indicators (eye and foot examination, microalbuminuria screening, blood cholesterol and glycated hemoglobin measurement [HbA1c], influenza immunization, blood pressure measurement, physical activity and diet advice) and outcome indicators (12-Item Short-Form Health Survey [SF-12], Audit of Diabetes-Dependent Quality of Life [ADDQoL], Patient Assessment of Chronic Illness Care [PACIC], Diabetes Self-Efficacy Scale, HbA1c value, and blood pressure <140/90 mmHg). For each profile of ambulatory healthcare use, we calculated adjusted probabilities of receiving processes of care and estimated adjusted outcomes of care using logistic and linear regression models, respectively.

**Results:**

Four profiles of ambulatory healthcare use were identified: participants with more visits to the general practitioner [GP] than to the diabetologist and receiving concomitant podiatry care (“GP & podiatrist”, n=86); participants visiting almost exclusively their GP (“GP only”, n=195); participants with a substantially higher use of all ambulatory services (“High users”, n=96); and participants reporting more visits to the diabetologist and less visits to the GP than other profiles (“Diabetologist first”, n=173). Whereas participants belonging to the “GP only” profile were less likely to report most processes related to the quality of diabetes care, outcomes of care were relatively comparable across all ambulatory healthcare use profiles.

**Conclusions:**

Slight differences in quality of diabetes care appear across the four ambulatory healthcare use profiles identified in this study. Overall, however, results suggest that room for improvement exists in all profiles, and further investigation is necessary to determine whether individual characteristics (like diabetes-related factors) and/or healthcare factors contribute to the differences observed between profiles.

## Introduction

The last decades have seen a steady increase in the prevalence of diabetes worldwide, which is also associated with a considerable human and economic burden (1). Healthcare systems have achieved mixed results in terms of quality and outcomes of diabetes care (2, 3). The relationship between the type of health services delivered to patients with diabetes and quality of care is complex and available evidence is equivocal. Some reports found that specialty care achieved better processes and outcomes than primary care (4–8), while others did not (9–11), or observed better process measures in patients with diabetes seen concomitantly by primary care and specialty physicians (12, 13). Moreover, an overview of systematic reviews highlighted the effectiveness of expanding the role of non-medical health professionals and using multidisciplinary teams to improve diabetes care (14).

In an attempt to meet the healthcare needs of patients with chronic diseases, there is an increasing interest in tools allowing the segmentation of populations into groups sharing common characteristics, such as health service utilization patterns (15, 16). For instance, data-driven clustering of a heterogeneous patient population based on age and healthcare use allowed the identification of distinct patient profiles with differing subsequent healthcare utilization and mortality in Singapore (17). In the field of diabetes care, population segmentation methods are also increasingly used (18, 19). Three profiles of diabetic patients with a distinct use of health services were identified in Dutch general practices using a data-driven segmentation approach (20). The first profile was characterized by high healthcare utilization and frequent home visits, the second by infrequent consultations limited to the general practitioner (GP), and the third by a high number of consultations with both GPs and primary care nurses. Patients belonging to these profiles differed in terms of age and type of diabetes medication. Another Australian study grouped patients with diabetes with similar patterns of GP utilization and showed that all groups had lower rates of diabetes-related preventable hospitalizations than those who did not consult a GP (21). Finally, a cluster analysis conducted in the United States Veterans Healthcare Administration showed that the majority of patients with diabetes had a low and decreasing use of primary care, and that this profile was associated with shorter survival compared to those with a consistent use of primary care over time (22). However, there is a paucity of literature exploring patterns of healthcare use in patients with diabetes and evidence regarding the association of such patterns with quality of diabetes care is lacking.

The primary objective of the present study was to identify profiles of patients with distinct ambulatory healthcare use patterns and describe the patient characteristics associated with these profiles. The secondary objective was to explore the association between these profiles and the quality of diabetes care, as measured by specific processes of care and outcome indicators.

## Materials and Methods

### Study Design and Participants

CoDiab-VD is a prospective, community-based, cohort study on diabetes care launched in 2011 in the canton of Vaud (French-speaking part of Switzerland) (23). During the two recruitment phases (2011-2012 and 2017), individuals visiting community-based pharmacies with a diabetes-related prescription were offered participation in the study. Inclusion criteria were non-institutionalized adults (≥18 years old) who had been diagnosed with diabetes for at least 12 months and living in the canton of Vaud. Women with gestational diabetes and individuals with severe cognitive impairment or without sufficient French language skills to complete the questionnaire were excluded. Eligible individuals accepting to participate were given a baseline paper questionnaire to be returned to investigators by regular mail. Participants were then followed annually by postal questionnaire. This study is based on self-reported cross-sectional data from the baseline questionnaire survey conducted during the two recruitment phases.

The Ethics Committee of Research on Human Beings of the Canton of Vaud approved the protocol of the CoDiab-VD cohort study (ID 151/11 and PB_2017_00232), and the protocol was registered on ClinicalTrials.gov (NCT01902043). All participants provided written informed consent. Our work is reported according to guidelines for observational studies developed by the STROBE initiative (24).

### Measurements

The baseline questionnaire encompassed different aspects of participants’ health status, diabetes care and daily life.

#### Use of Ambulatory Healthcare

We considered the number of visits to the following ambulatory healthcare providers in the past 12 months to identify profiles of patients with distinct use patterns: GP; diabetologist; diabetes nurse; dietitian; podiatrist; and emergency visit to a doctor’s office or an emergency department (ordinal variables: never, once, 2-3 times, 4 times and more). In Switzerland, podiatrists are allied health professionals who specialize in the diagnosis and nonsurgical treatment of disorders of the foot.

#### Quality of Diabetes Care

We used both processes of care and outcome indicators to measure the quality of diabetes care. Process variables were binary (i.e. the process was reported or not) and referred to the past 12 months, unless otherwise specified. Included processes were foot examination, microalbuminuria screening, blood cholesterol measurement, influenza immunization, and eye examination (in the past 24 months). Similar to previous research (25), we created two binary composite variables from these processes, i.e. receipt of at least four of the five processes (yes/no) and receipt of all five processes (yes/no). Participants with knowledge of glycated hemoglobin (HbA1c) reported if they had had ≥2 measurements of HbA1c in the past 12 months and we created analogous composite variables including this process (i.e. receipt of at least five of the six processes and receipt of all six processes). We also considered two additional process variables, i.e. reporting of ≥2 blood pressure (BP) measurements and physical activity and diet advice in the past 12 months. We evaluated diabetes care outcomes using the following validated instruments: the 12-item Short-Form Health Survey (SF-12) for general health status with both the physical component summary (PCS) and the mental component summary (MCS) (range for each summary score, 0 to 100) (26); the 19-item Audit of Diabetes-Dependent Quality of Life (ADDQoL; score range, -9 to +3) (27); the 20-item Patient Assessment of Chronic Illness Care (PACIC) for evaluation of patient-centered care (score range, 1 to 5) (28, 29); and the Diabetes Self-Efficacy Scale (score range, 1 to 10; only participants of the 2017 recruitment phase) (30). Additionally, we included as outcome indicators the last value of HbA1c (continuous) and whether the last BP measurement was below 140/90 mmHg (yes/no).

#### Independent Variables

Independent variables included in this study were as follows. Sociodemographic characteristics: age (continuous); sex (female, male); living arrangement status (alone, with ≥1 person); residential location (urban, intermediary, rural); education level (primary, secondary, tertiary); quarter of household income (reference: canton of Vaud); mandatory health insurance model (standard or alternative, e.g. Health Maintenance Organization); and receipt of subsidies for mandatory health insurance (yes/no). Diabetes status and management: type of diabetes (type 1, type 2, other or unknown); duration of known diabetes (1-10 years, >10 years); antidiabetic medication (including or excluding insulin or other injectable); number of diabetes-related complications (continuous); self-monitoring of blood glucose (yes/no); knowledge of HbA1c (yes/no); participation in diabetes education courses (yes/no); and membership in local diabetes association (yes/no). Health status and habits: self-rated health as measured by the first question of the SF-12 (26); number of comorbidities (continuous); body mass index category; smoking (former or non-smoker, current smoker); physical activity (active, partially active, inactive); and screening for depression using a two-question case-finding instrument (31). Health services utilization: hospitalization in the past 12 months (none, once, more than once), forgoing care due to cost in the past 12 months (yes/no).

### Statistical Analyses

As a first step, we considered visits to the GP, diabetologist, diabetes nurse, dietitian, podiatrist, and emergency visits to identify clusters of individuals with similar profiles of ambulatory healthcare use. We investigated several model-based clustering methods to identify clusters (32–34). We fitted discrete mixture models based on Poisson and ordinal logit distributions to the counts of the six variables considered (coded 0 for “never”, 1 for “once”, 2 for “2-3 times”, and 4 for “4 times and more”). Due to convergence issues with the ordinal logit distributions, the mixture of Poisson distributions was finally retained. We also considered a slightly different coding scheme as a sensitivity analysis (0 for “never”, 1 for “once”, 3 for “2-3 times”, and 4 for “4 times or more”), which produced comparable results. To select the number of clusters, we used the Bayesian Information Criterion (BIC) and examined how observations were reassigned to clusters as the number of clusters was increased using a clustergram. After convergence of the clustering algorithm, we plotted the mean number of visits in each cluster to visualize the profiles identified. To calculate the means, the original ordinal variables were recoded as follows: 0 for “never”, 1 for “once”, 2.5 for “2-3 times”, and 4 for “4 times or more”. Since the clustering method could not handle observations that had missing data, we excluded individuals with ≥1 missing value in the six variables considered for clustering. No missing data imputations were performed as it was unclear from a statistical point of view how to handle multiple imputations with clustering algorithms.

In a second phase, we proceeded to a description of participant characteristics and evaluated the statistical significance of differences between healthcare use profiles using the Kruskal–Wallis test for continuous variables and the chi-square test for categorical variables. For each healthcare use profile, we estimated the probabilities of receiving recommended processes of care and their 95% confidence intervals (CI). To that end, we performed crude and adjusted logistic regression models and computed predictive margins. We included age, sex, living arrangement status, residential location, education level, mandatory health insurance model, subsidies for mandatory health insurance, and diabetes-related complications in adjusted models. Finally, we estimated mean scores of the different outcomes of care and their 95% CI for each healthcare use profile using crude and adjusted linear regression models (same adjustment variables). We estimated the probability of a BP measurement <140/90 mmHg using a logistic regression model given the dichotomous nature of the variable. Statistical analyses were performed using Stata/IC 16.1 (StataCorp, College Station, TX, USA).

## Results

Of the 1033 individuals who answered the self-administered baseline questionnaire (519 in 2011-12 and 514 in 2017), 483 were excluded because of missing values in the variables considered for clustering. A total of 550 participants aged 19 to 92 years were included in the final analyses (male, 58.2%; **Table 1**). Most participants (71.8%) had type 2 diabetes.

**Table 1:** Characteristics of participants, by healthcare use profile.

	All (N=550)		Profile 1 “GP & podiatrist” (N=86)		Profile 2 “GP only” (N=195)		Profile 3 “High users” (N=96)		Profile 4 “Diabetologist first” (N=173)		p-value
	% or mean (SD, min-max)	(n)	% or mean (SD, min-max)	(n)	% or mean (SD, min-max)	(n)	% or mean (SD, min-max)	(n)	% or mean (SD, min-max)	(n)	
**Sociodemographic characteristics**											
**Age (years)**	62.1 (13.5, 19-92)	(550)	69.6 (11.3, 29-92)	(86)	65.8 (10.3, 28-87)	(195)	60.7 (13.6, 22-88)	(96)	55.1 (14.4, 19-84)	(173)	<0.001
<65	51.8	(285)	33.7	(29)	40.0	(78)	58.3	(56)	70.5	(122)	
65-74	31.3	(172)	29.1	(25)	41.0	(80)	27.1	(26)	23.7	(41)	
≥75	16.9	(93)	37.2	(32)	19.0	(37)	14.6	(14)	5.8	(10)	
**Sex**											0.003
Female	41.8	(230)	48.8	(42)	31.8	(62)	51.0	(49)	44.5	(77)	
Male	58.2	(320)	51.2	(44)	68.2	(133)	49.0	(47)	55.5	(96)	
**Living arrangement status**											0.023
Alone	28.6	(157)	30.2	(26)	23.1	(45)	40.4	(38)	27.7	(48)	
With ≥1 person	71.4	(391)	69.8	(60)	76.9	(150)	59.6	(56)	72.3	(125)	
**Residential location**											0.135
Urban	64.9	(355)	65.9	(56)	62.4	(121)	56.3	(54)	72.1	(124)	
Intermediary	20.5	(112)	23.5	(20)	20.1	(39)	26.0	(25)	16.3	(28)	
Rural	14.6	(80)	10.6	(9)	17.5	(34)	17.7	(17)	11.6	(20)	
**Education level**											0.162
Primary	15.5	(82)	12.9	(11)	15.5	(29)	18.4	(16)	15.4	(26)	
Secondary	53.8	(284)	62.4	(53)	56.2	(105)	55.2	(48)	46.2	(78)	
Tertiary	30.7	(162)	24.7	(21)	28.3	(53)	26.4	(23)	38.5	(65)	
**Household income**											0.090
<1st quartile	22.7	(110)	28.6	(22)	23.0	(41)	29.5	(23)	15.9	(24)	
1st quartile - <2nd quartile	29.6	(143)	26.0	(20)	31.5	(56)	29.5	(23)	29.1	(44)	
2nd quartile - <3rd quartile	31.6	(153)	31.2	(24)	32.6	(58)	30.8	(24)	31.1	(47)	
≥3rd quartile	16.1	(78)	14.3	(11)	12.9	(23)	10.3	(8)	23.8	(36)	
**Mandatory health insurance model**											0.507
Standard	72.3	(388)	74.1	(63)	69.1	(132)	77.4	(72)	72.0	(121)	
Alternative (e.g. HMO, medical helpline first)	27.7	(149)	25.9	(22)	30.9	(59)	22.6	(21)	28.0	(47)	
**Subsidies for mandatory health insurance**											0.214
Yes	20.0	(107)	19.0	(16)	16.8	(32)	27.5	(25)	20.2	(34)	
No	80.0	(427)	81.0	(68)	83.2	(159)	72.5	(66)	79.8	(134)	
**Diabetes status and management**											
**Type of diabetes**											<0.001
Type 1	13.8	(76)	8.1	(7)	2.6	(5)	18.8	(18)	26.6	(46)	
Type 2	71.8	(395)	80.2	(69)	74.4	(145)	76.0	(73)	62.4	(108)	
Other or does not know	14.4	(79)	11.6	(10)	23.1	(45)	5.2	(5)	11.0	(19)	
**Duration of known diabetes**											<0.001
1-10 years	49.0	(267)	38.8	(33)	63.0	(121)	49.0	(47)	38.4	(66)	
>10 years	51.0	(278)	61.2	(52)	37.0	(71)	51.0	(49)	61.6	(106)	
**Antidiabetic medication**											<0.001
Excluding insulin or other injectable	46.5	(255)	36.5	(31)	77.4	(151)	25.0	(24)	28.5	(49)	
Including insulin or other injectable	53.5	(293)	63.5	(54)	22.6	(44)	75.0	(72)	71.5	(123)	
Number of diabetes-related complications*	0.6 (0.9, 0-5)	(544)	0.7 (0.9, 0-4)	(85)	0.4 (0.7, 0-3)	(192)	1.0 (1.2, 0-5)	(95)	0.6 (0.9, 0-5)	(172)	<0.001
**Self-monitoring of blood glucose**											<0.001
Yes	82.6	(451)	89.4	(76)	64.9	(126)	95.7	(90)	91.9	(159)	
No	17.4	(95)	10.6	(9)	35.1	(68)	4.3	(4)	8.1	(14)	
**Has already heard about HbA1c**											<0.001
Yes	78.1	(389)	78.2	(61)	64.1	(109)	89.7	(78)	86.5	(141)	
No	21.9	(109)	21.8	(17)	35.9	(61)	10.3	(9)	13.5	(22)	
**Participation in diabetes education courses**											<0.001
Yes	34.1	(185)	38.8	(33)	17.0	(33)	46.2	(43)	44.4	(76)	
No	65.9	(358)	61.2	(52)	83.0	(161)	53.8	(50)	55.6	(95)	
**Member of the local diabetes association**											<0.001
Yes	14.1	(76)	21.2	(18)	3.6	(7)	23.7	(22)	17.3	(29)	
No	85.9	(462)	78.8	(67)	96.4	(185)	76.3	(71)	82.7	(139)	
**Health status and habits**											
**Self-rated health**											0.192
Excellent	2.2	(12)	2.4	(2)	2.6	(5)	1.1	(1)	2.3	(4)	
Very good	13.4	(72)	12.1	(10)	14.2	(27)	9.6	(9)	15.1	(26)	
Good	61.4	(331)	60.2	(50)	64.2	(122)	54.3	(51)	62.8	(108)	
Fair	18.9	(102)	21.7	(18)	16.8	(32)	25.5	(24)	16.3	(28)	
Poor	4.1	(22)	3.6	(3)	2.1	(4)	9.6	(9)	3.5	(6)	
**Number of comorbidities°**	1.7 (1.3, 0-6)	(538)	2.1 (1.5, 0-6)	(83)	1.8 (1.3, 0-5)	(192)	2.0 (1.5, 0-6)	(92)	1.4 (1.2, 0-6)	(171)	<0.001
**Body mass index**											0.407
Underweight (<18.5 kg/m2)	0.8	(4)	0.0	(0)	0.5	(1)	2.2	(2)	0.6	(1)	
Normal (18.5-24.9 kg/m2)	18.6	(97)	19.3	(16)	15.1	(28)	17.8	(16)	22.7	(37)	
Overweight (25-29.9 kg/m2)	35.8	(187)	31.3	(26)	39.8	(74)	31.1	(28)	36.2	(59)	
Obese (≥30 kg/m2)	44.8	(234)	49.4	(41)	44.6	(83)	48.9	(44)	40.5	(66)	
**Smoking**											0.452
Former or non-smoker	81.9	(444)	86.9	(73)	81.3	(157)	83.9	(78)	79.1	(136)	
Current smoker	18.1	(98)	13.1	(11)	18.7	(36)	16.1	(15)	20.9	(36)	
**Physical activity†**											0.210
Active	53.7	(289)	47.6	(39)	50.5	(98)	64.5	(60)	54.4	(92)	
Partially active	18.8	(101)	20.7	(17)	19.1	(37)	11.8	(11)	21.3	(36)	
Inactive	27.5	(148)	31.7	(26)	30.4	(59)	23.7	(22)	24.3	(41)	
**Positive screening for depression**											0.087
Yes	34.7	(188)	33.7	(28)	30.1	(58)	45.3	(43)	34.5	(59)	
No	65.3	(354)	66.3	(55)	69.9	(135)	54.7	(52)	65.5	(112)	
**Health services utilization**											
**Hospitalization in the past 12 months**											0.005
None	76.4	(415)	76.7	(66)	79.4	(154)	64.5	(60)	79.4	(135)	
Once	15.7	(85)	12.8	(11)	16.5	(32)	18.3	(17)	14.7	(25)	
More than once	7.9	(43)	10.5	(9)	4.1	(8)	17.2	(16)	5.9	(10)	
**Has forgone care due to cost in the past 12 months**											0.017
Yes	16.7	(91)	20.9	(18)	10.3	(20)	16.7	(16)	22.0	(37)	
No	83.3	(453)	79.1	(68)	89.7	(174)	83.3	(80)	78.0	(131)	

Statistical significance of differences between healthcare use profiles calculated using the Kruskal–Wallis test for continuous variables and the chi-square test for categorical variables.

GP, general practitioner; SD, standard deviation; HMO, Health Maintenance Organization; HbA1c, glycated hemoglobin.

*Among the following: ischemic heart disease, stroke, retinopathy, chronic kidney disease without dialysis, chronic kidney disease with dialysis or kidney transplant, neuropathy, foot ulcer, lower limb amputation, severe hypo- or hyperglycemia.

°Among the following: heart disease, chronic lung disease, osteoporosis, osteoarthritis or arthritis, malignancy, gastric or duodenal ulcer, depression, Parkinson’s disease, hypertension, hypercholesterolemia, other chronic condition.

†Active: ≥150 minutes of moderate physical activity or ≥2 intense activities per week; partly active: 30 to 149 minutes of moderate physical activity or 1 intense activity per week; inactive: <30 minutes of moderate physical activity and <1 intense activity per week.

### Characteristics of Ambulatory Healthcare Use Profiles

**Figure 1:**
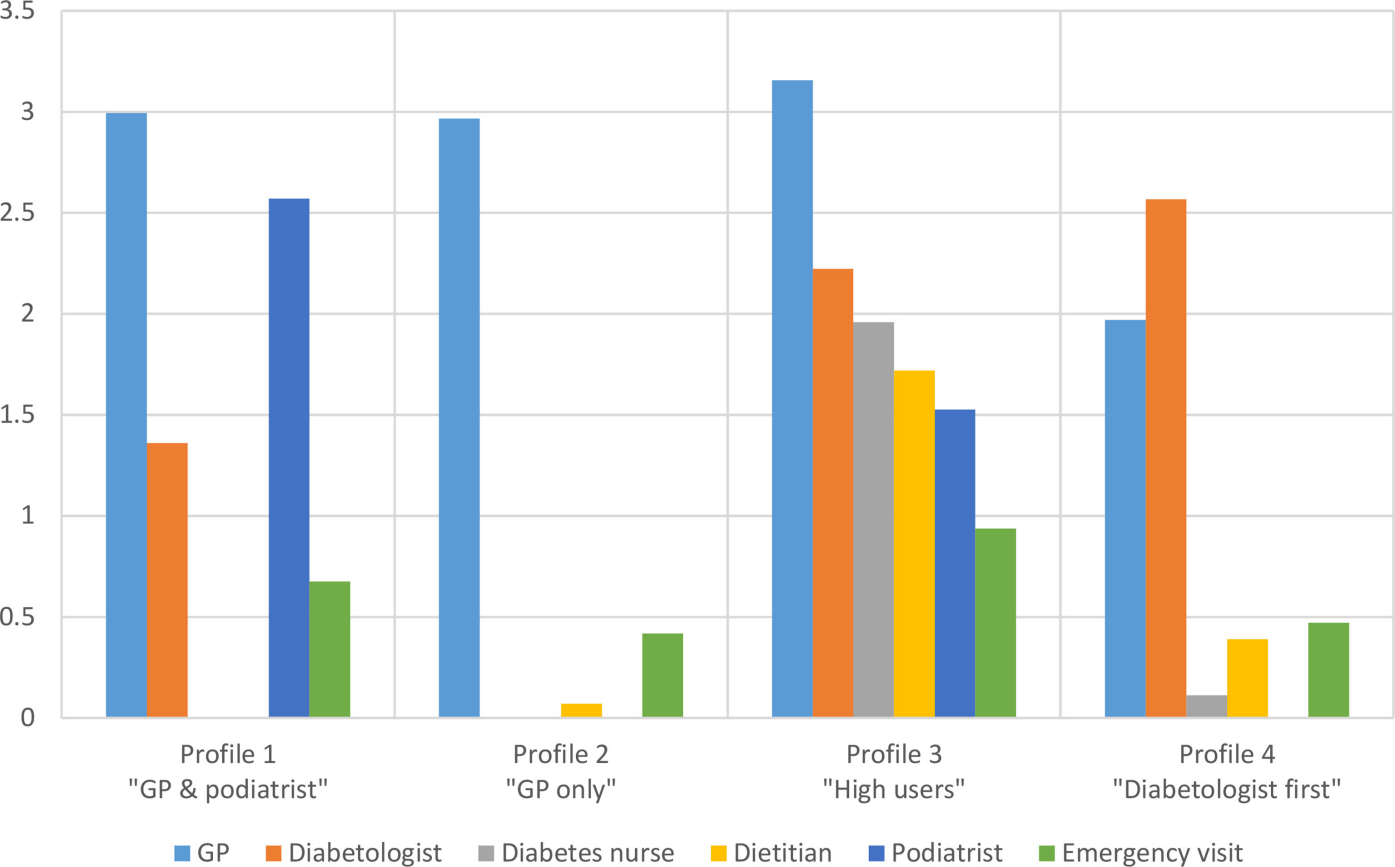
Mean number of visits in the past 12 months, by provider and healthcare use profile. GP, general practitioner.

The mixture model selected on the basis of the BIC and the clustergram identified four distinct ambulatory care use profiles (**Figure 1**). Profile 1 (“GP & podiatrist”) was characterized by more frequent visits to the GP than to the diabetologist, and a higher use of podiatry than other profiles (n=86). Participants belonging to profile 2 (“GP only”) reported visiting almost exclusively their GP (n=195). Profile 3 (“High users”) included participants characterized by a substantially higher use of all ambulatory services than other profiles (n=96). It encompassed the majority of participants reporting visits to a diabetes nurse and dietician and was characterized by a wider use of emergency visits. Participants belonging to profile 4 (“Diabetologist first”) reported more visits to the diabetologist than other profiles and a lower use of GP services (n=173).

**Table 1** presents the characteristics of the individuals belonging to the four profiles. Participants were younger in the “Diabetologist first” profile and older in the “GP & podiatrist” profile. Male participants (68.2%) were overrepresented in the “GP only” profile, whereas the sex ratio was more balanced in other profiles. The share of participants living alone was higher in the “High users” profile. Regarding diabetes characteristics, participants with type 1 diabetes were substantially represented in the “Diabetologist first” (26.6%) and “High users” (18.8%) profiles. In the “GP only” profile, the proportion of participants with a duration of diabetes >10 years (37.0%) and using insulin or other injectable (22.6%) was lower than in other profiles (51.0% to 61.6%, and 63.5% to 75.0%, respectively). The mean number of diabetes-related complications was highest in the “High users” profile and lowest in the “GP only” profile. In the latter profile, the share of participants who reported self-monitoring of blood glucose and had already heard about HbA1c was lower than in other profiles. The proportion of individuals reporting participation in diabetes education courses and membership in local diabetes association was also lower in this profile. The mean number of comorbidities was lower in the “Diabetologist first” profile. More participants reported ≥1 hospitalization in the past 12 months in the “High users” profile (35.5%) than in other profiles (20.6% to 23.3%). The proportion of participants reporting that they had forgone care due to cost was lower in the “GP only” profile.

### Association Between Ambulatory Healthcare Use Profiles and the Quality of Diabetes Care

**Figure 2:**
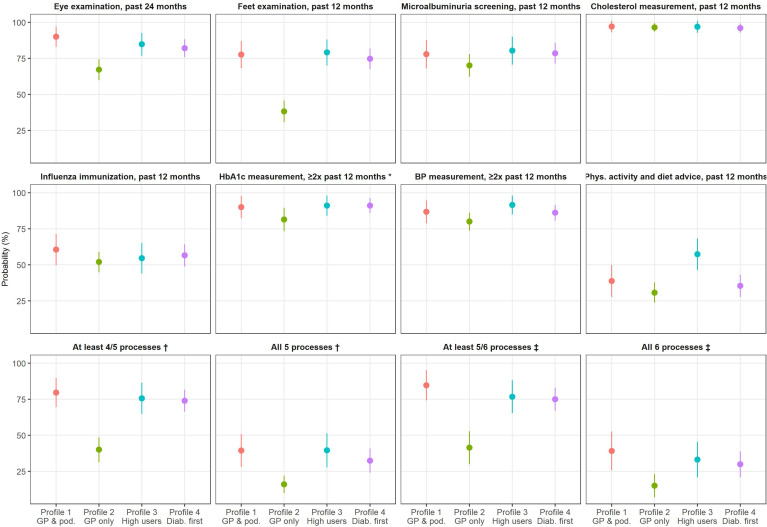
Adjusted probabilities of receiving recommended processes of care, by healthcare use profile. GP, general practitioner. HbA1c, glycated hemoglobin. BP, blood pressure. Probabilities estimated from logistic regression models (predictive margins). Adjustment: age, sex, living arrangement status, residential location, education level, mandatory health insurance model, subsidies for mandatory health insurance, and diabetes-related complications. *Only participants who have already heard about HbA1c. †Among the following: eye examination, foot examination, microalbuminuria screening, blood cholesterol measurement, and influenza immunization. ‡Among the following: eye examination, foot examination, microalbuminuria screening, blood cholesterol measurement, influenza immunization, and HbA1c measurement.

Adjusted probabilities of receiving recommended processes of care are shown in **Figure 2** (exact figures are provided in **Supplementary Table 1**). Participants in the “GP only” profile were less likely to report an eye examination in the past 24 months (67.2%) than those belonging to other profiles (82.1% to 90.0%). The gap was even more pronounced for foot examination in the past 12 months (38.2% in the “GP only” profile; 74.6% to 79.1% in other profiles). Use of laboratory tests in the past 12 months was more homogeneous across profiles (70.1% to 80.3% for microalbuminuria, 96.0% to 97.1% for blood cholesterol, and 81.4% to 91.1% for HbA1c), as was the administration of influenza immunization (51.9% to 60.5%) and BP measurement (80.0% to 91.5%). The probability of receiving physical activity and diet advice in the past 12 months was higher in the “High users” profile. Regarding composite variables, the probability of receiving at least 4/5 processes of care was approximately 75% in all profiles, apart from “GP only” (40.0%). Similar figures were found for the achievement of 5/6 processes. However, the probabilities of receiving all five or six processes were much lower in all profiles, although still below in the “GP only” profile (approximately 15%, compared to 30-40% in other profiles).

**Figure 3:**
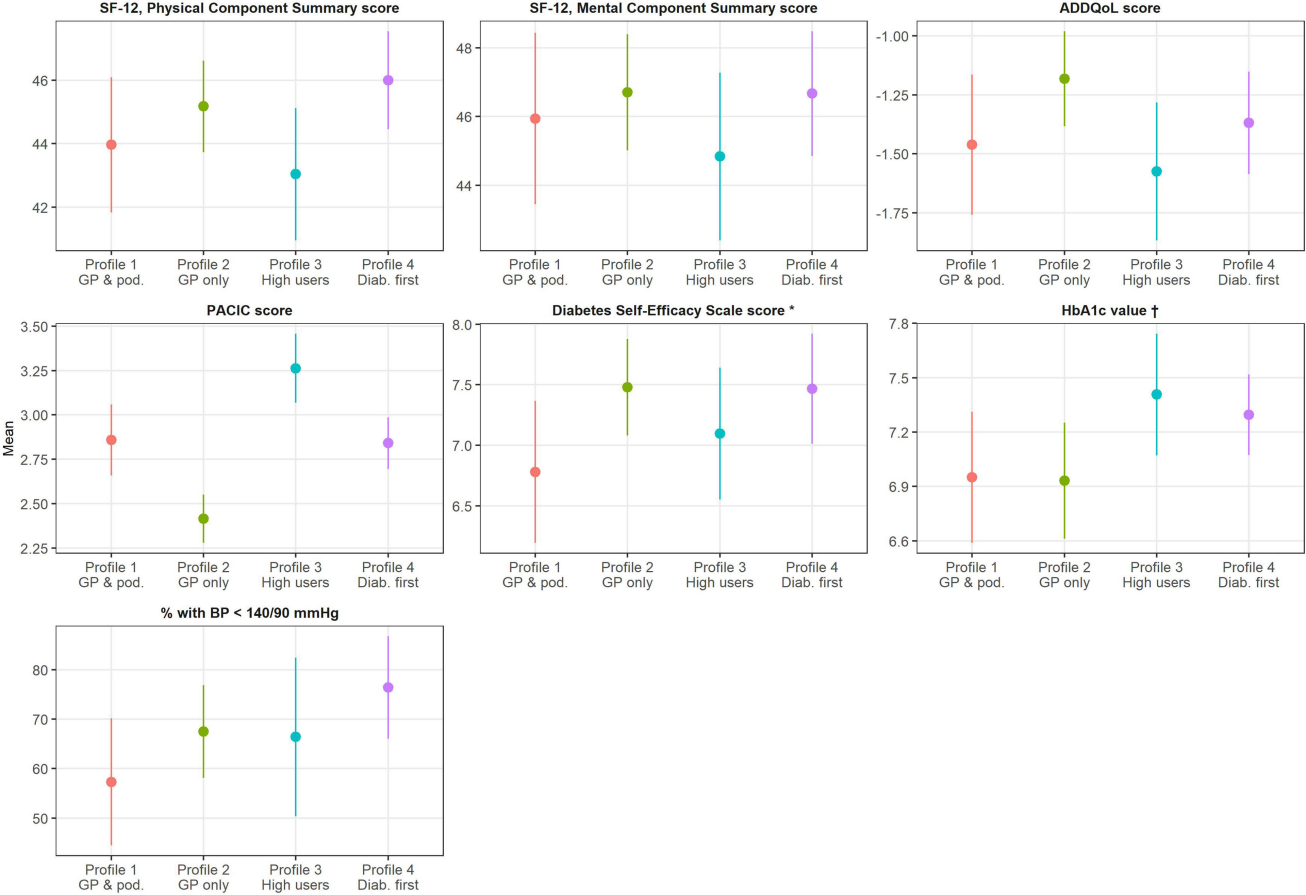
Adjusted outcomes of care, by healthcare use profile. GP, general practitioner; SF-12, 12-Item Short-Form Health Survey; ADDQoL, Audit of Diabetes-Dependent Quality of Life; PACIC, Patient Assessment of Chronic Illness Care; HbA1c, glycated hemoglobin; BP, blood pressure. Means (or probabilities) estimated from linear (or logistic) regression models (predictive margins). Adjustment: age, sex, living arrangement status, residential location, education level, mandatory health insurance model, subsidies for mandatory health insurance, and diabetes-related complications. *Only participants of the 2017 recruitment phase. † Only participants who have already heard about HbA1c.

Adjusted outcomes of care are shown in **Figure 3** (exact figures are provided in **Supplementary Table 2**). The SF-12 PCS and MCS mean scores were comparable across profiles, as was the mean score of the ADDQoL. Regarding the PACIC, participants in the “High users” profile had a higher mean score (3.3) and those in the “GP only” profile a lower mean score (2.4) than participants in the “GP & podiatrist” and “Diabetologist first” profiles (2.9 and 2.8, respectively). Mean scores of the Diabetes Self-Efficacy Scale were quite homogeneous across profiles. Mean HbA1c values tended to be lower in the “GP & podiatrist” and “GP only” profiles (7.0% and 6.9%, respectively) than those in the “High users” and “Diabetologist first” profiles (7.4% and 7.3%, respectively). Probability of a BP measurement <140/90 mmHg varied between 57.3% in the “GP & podiatrist” profile and 76.4% in the “Diabetologist first” profile.

## Discussion

In this community-based sample of patients with diabetes, we identified four distinct and clinically meaningful profiles of ambulatory healthcare use: participants with more visits to the GP than to the diabetologist and receiving concomitantly podiatry care (“GP & podiatrist”); participants visiting almost exclusively their GP (“GP only”); participants with a substantially higher use of all ambulatory services (“High users”); and participants reporting more visits to the diabetologist and less visits to the GP than other profiles (“Diabetologist first”). Sociodemographic characteristics of participants varied between profiles, as did diabetes characteristics and management. Whereas participants belonging to the “GP only” profile were less likely to report most processes related to the quality of diabetes care, outcomes of care were quite comparable across ambulatory healthcare use profiles.

### Characteristics of Ambulatory Healthcare Use Profiles

Most participants in the “GP only” profile reported a duration of diabetes of <10 years and took only oral hypoglycemic agents. The number of diabetes-related complications was also lower in this profile. These factors might indicate less advanced disease, which could partly explain the almost exclusive reliance on GP services and the poorer self-management skills reported by these participants. Similarly, a Dutch study using a data-driven segmentation approach showed that patients with diabetes characterized by consultations that were infrequent and limited to the GP had a less intensive anti-diabetic treatment than those with higher healthcare utilization (20).

The share of participants reporting a longer duration of diabetes and the use of insulin or other injectable was substantially higher in the “GP & podiatrist” profile than in the “GP only” one, which could explain the involvement of the diabetologist concomitantly with the GP. Additionally, the older age of participants in the “GP & podiatrist” profile is congruent with findings from a recent study, which showed that patients referred to the podiatrist by their GP were more likely to be aged ≥85 years (35). Moreover, older age and a longer duration of diabetes are known risk factors for foot ulceration and the higher use of podiatry may also reflect an increased risk of diabetic foot in this profile (36).

Participants in the “Diabetologist first” profile were more likely to have type 1 diabetes, which could contribute to the prevalence of specialty care in this profile. Contrary to previous research reporting higher rates of potentially preventable hospitalizations in patients with diabetes not seeing a GP (21), participants in the “Diabetologist first” profile did not report a higher amount of emergency visits or hospitalizations, despite a lower use of GP services than other profiles. This probably reflects a complex relationship between the use of ambulatory healthcare and hospitalization, the latter being also influenced by factors such as diabetes-related morbidity (37).

Finally, participants in the “High users” profile combined health and socioeconomic vulnerabilities. This association of the comorbidity burden and material need insecurities with the use of health services in patients with diabetes has been previously reported (20, 38, 39). Additionally, the complexity of care needs in this group of highly comorbid and vulnerable patients was reflected by multidisciplinary care, which was particularly prevalent in this profile.

### Association Between Ambulatory Healthcare Use Profiles and the Quality of Diabetes Care

The proportion of participants reporting an eye examination was higher than previously reported in studies conducted in Switzerland and comparable to most countries included in a European cross-sectional study on the quality of diabetes care (3, 40–42). However, the comparison is limited due to a different timeframe in these studies (i.e. 12 months versus 24 months in our study). Regarding other processes of care, Stone et al. showed high between-country variation in the prevalence of foot examination (47.8% to 89.5%) and microalbuminuria screening (26.7% to 90.3%) in European countries (3). Our observations are close to those of high-ranking countries, apart from the prevalence of foot examination in the “GP only” profile, which is substantially lower than European findings. We found that the proportion of participants reporting HbA1c, blood cholesterol and BP measurement was >80%, which is consistent with observations made in Europe, but higher than previous findings in Switzerland, especially regarding cholesterol testing (3, 40–43). The prevalence of influenza immunization in our sample is comparable to observations recently reported in Spain, the United Kingdom and the USA (44–46). Our results confirm that processes of care included in composite indicators are rarely all fulfilled as reported in a Swiss study showing that only 17.2% of the participants achieved the six quality indicators used to assess performance (43). The lower prevalence of several processes of care in the “GP only” profile seems to corroborate past research, which found that specialty care, alone or in addition to primary care, achieved a better quality than primary care alone (4–8, 12, 13). However, as mentioned earlier, participants in the “GP only” profile had less advanced disease, which could also have influenced the preventive care received.

Regarding care outcomes, participants in the “GP only” profile tended to report higher scores in indicators related to general health status (PCS and MCS), quality of life (ADDQoL) and self-efficacy, similar to participants in the “Diabetologist first” profile. As shown in previous studies, this suggests that processes of care and these outcome indicators are not consistently associated (25). The fact that participants in the “GP & podiatrist” and “High users” profiles tended to report lower scores in the same outcome indicators may be related to their older age and higher comorbidity burden, respectively. Despite statistical adjustment, it remains difficult at present to determine the respective contributions of individual health and diabetes-related factors to the quality of diabetes care on the one hand, and healthcare factors on the other hand. Interestingly, despite combining health and socioeconomic vulnerabilities, care delivered to participants in the “High users” profile was more congruent with the chronic care model (28), as reflected by higher PACIC scores. This might be related to the provision of multidisciplinary care in this profile. By contrast, participants in the “GP only” profile reported lower PACIC scores, which corroborates the findings of a systematic review (47). Regarding mean HbA1c values, our observations are congruent with the findings of Stone et al. in Europe (mean values ranging from 6.8% to 7.5%) (3). Values tended to be higher in profiles with a greater proportion of participants with type 1 diabetes and the use of insulin (i.e. “High users” and “Diabetologist first” profiles), but this must be interpreted with caution given the magnitude of missing data.

### Strengths and Limitations

The main strength of this study is the use of reliable clustering methods, which allowed to identify profiles of patients with consistent characteristics and distinct ambulatory healthcare use patterns. To our knowledge, this study is the first to have examined the association between healthcare use profiles identified using a data-driven segmentation approach and the quality of diabetes care. Another strength lies in the fact that we included outcome indicators rarely reported in the literature published so far, such as patient-reported experiences of care (48). However, the results of this study need to be interpreted considering the following three main limitations. First, we had to exclude a substantial number of individuals from the analyses due to missing values in the variables considered for clustering. This may have limited our ability to detect differences in the characteristics of ambulatory healthcare use profiles and their association with the quality of diabetes care. Second, the validity of collected data may be limited as they were self-reported. However, there is no evidence suggesting that such a limitation would have differentially affected participants’ responses and introduced bias. Third, the generalizability of our results to other settings may be limited by specificities of the Swiss healthcare system. For instance, visits to specialist physicians occur most of the time outside hospital, in private practices, which is not the case in other countries.

## Conclusions

In summary, patients with diabetes can be categorized into four distinct and clinically meaningful ambulatory healthcare use patterns intricately associated with sociodemographic, health and diabetes characteristics. Quality of care results measured by specific processes of care and outcome indicators appear to differ across ambulatory healthcare use profiles and suggest that room for improvement exists in all profiles. However, the respective contributions of individual characteristics and healthcare factors to the differences in quality of care observed between profiles remain unclear and warrant further research.

## Data Availability Statement

The datasets presented in this study can be found in online repositories. The names of the repository/repositories and accession number(s) can be found below: https://doi.org/10.16909/dataset/18.

## Ethics Statement

The studies involving human participants were reviewed and approved by the Ethics Committee of Research on Human Beings of the Canton of Vaud. The patients/participants provided their written informed consent to participate in this study.

## Author Contributions

Concept and design: IP-B. Data acquisition: EZ and IP-B. Statistical analysis: JD, EZ, and PT. Interpretation of data: all authors. Drafting of the manuscript: JD, EZ, and PT. Critical revision of the manuscript for important intellectual content: all authors. All authors contributed to the article and approved the submitted version.

## Funding

The CoDiab-VD cohort study was supported by the Department of Health and Social Action, Canton of Vaud. The sponsors had no role in the collection, analysis and interpretation of the data, nor in the writing of the report and the decision to submit the paper for publication. Open access funding was provided by the University of Lausanne.

## Conflict of Interest

The authors declare that the research was conducted in the absence of any commercial or financial relationships that could be construed as a potential conflict of interest.

## Publisher’s Note

All claims expressed in this article are solely those of the authors and do not necessarily represent those of their affiliated organizations, or those of the publisher, the editors and the reviewers. Any product that may be evaluated in this article, or claim that may be made by its manufacturer, is not guaranteed or endorsed by the publisher.
