# Erratum: Ambulatory healthcare use profiles of patients with diabetes and their association with quality of care: A cross-sectional study

**DOI:** 10.3389/fendo.2023.1187133

**Published:** 2023-03-27

**Authors:** Frontiers Production Office

**Affiliations:** Frontiers Media SA, Lausanne, Switzerland

**Keywords:** diabetes mellitus, ambulatory care, profiles, cluster analysis, quality of health care, process assessment, outcome assessment

An omission to the funding section of the original article was made in error. The following sentence has been added: “Open access funding was provided by the University of Lausanne”.

The original version of this article has been updated.

