# Supplementary material for: Ambulatory Healthcare Use Profiles of Patients With Diabetes and Their Association With Quality of Care: A Cross-Sectional Study
Source: Front Endocrinol (Lausanne). 2022 Apr 13;13:841774. doi: 10.3389/fendo.2022.841774 (PMC9043606; doi:10.3389/fendo.2022.841774)
Supplement: Supplementary file 1 [file Table_1.docx]

**Supplementary Table 1.** Estimated probabilities of receiving recommended processes of care, by healthcare use profile

|  |  | **Profile 1 “GP & podiatrist”** | | **Profile 2 “GP only”** | | **Profile 3 “High users”** | | **Profile 4 “Diabetologist first”** | |
| --- | --- | --- | --- | --- | --- | --- | --- | --- | --- |
|  |  | Crude prob. [95% CI] | Adjusted prob. [95% CI] | Crude prob. [95% CI] | Adjusted prob. [95% CI] | Crude prob. [95% CI] | Adjusted prob. [95% CI] | Crude prob. [95% CI] | Adjusted prob. [95% CI] |
| Eye examination, in the past 24 months | N=491 | 91.4% [85.2% - 97.5%] | 90.0% [82.9% - 97.0%] | 67.1% [60.0% - 74.1%] | 67.2% [60.0% - 74.4%] | 85.2% [77.4% - 92.9%] | 84.7% [76.8% - 92.7%] | 80.8% [74.6% - 87.0%] | 82.1% [76.0% - 88.1%] |
| Foot examination, in the past 12 months | N=488 | 77.5% [68.3% - 86.7%] | 77.6% [68.3% - 87.0%] | 38.6% [31.3% - 45.9%] | 38.2% [30.8% - 45.7%] | 78.8% [69.8% - 87.7%] | 79.1% [70.1% - 88.0%] | 74.5% [67.7% - 81.3%] | 74.6% [67.5% - 81.7%] |
| Microalbuminuria screening, in the past 12 months | N=422 | 76.1% [66.1% - 86.0%] | 77.9% [68.1% - 87.6%] | 69.1% [61.4% - 76.7%] | 70.1% [62.3% - 77.9%] | 81.2% [71.9% - 90.4%] | 80.3% [70.7% - 90.0%] | 79.7% [73.1% - 86.3%] | 78.5% [71.2% - 85.7%] |
| Blood cholesterol measurement, in the past 12 months | N=479 | 97.5% [94.1% - 100.9%] | 97.1% [93.1% - 101.1%] | 96.4% [93.6% - 99.2%] | 96.5% [93.6% - 99.3%] | 97.3% [93.7% - 101.0%] | 96.9% [92.8% - 101.1%] | 95.5% [92.3% - 98.8%] | 96.0% [92.9% - 99.1%] |
| Influenza immunization, in the past 12 months | N=491 | 66.3% [55.9% - 76.6%] | 60.5% [49.8% - 71.3%] | 54.3% [46.9% - 61.7%] | 51.9% [44.8% - 59.1%] | 54.4% [43.4% - 65.4%] | 54.6% [44.0% - 65.2%] | 51.0% [43.1% - 58.8%] | 56.5% [48.8% - 64.3%] |
| At least 4/5 processes † | N=400 | 80.9% [71.5% - 90.2%] | 79.6% [69.4% - 89.7%] | 40.0% [31.6% - 48.4%] | 40.0% [31.3% - 48.7%] | 76.6% [66.2% - 86.9%] | 75.5% [64.7% - 86.4%] | 72.5% [65.0% - 79.9%] | 73.9% [66.3% - 81.5%] |
| All 5 processes † | N=400 | 42.6% [30.9% - 54.4%] | 39.4% [28.0% - 50.8%] | 17.7% [11.1% - 24.3%] | 15.9% [9.9% - 21.9%] | 39.1% [27.1% - 51.0%] | 39.6% [27.7% - 51.4%] | 28.3% [20.7% - 35.8%] | 32.4% [24.1% - 40.7%] |
| HbA1c measurement, ≥2x in the past 12 months * | N=347 | 89.7% [81.8% - 97.5%] | 90.0% [82.3% - 97.7%] | 81.1% [73.2% - 88.9%] | 81.4% [73.3% - 89.5%] | 90.9% [84.0% - 97.8%] | 91.0% [84.0% - 97.9%] | 91.4% [86.6% - 96.3%] | 91.1% [85.9% - 96.3%] |
| At least 5/6 processes ‡ | N=288 | 85.4% [75.4% - 95.4%] | 84.6% [74.2% - 95.0%] | 40.3% [28.9% - 51.6%] | 41.4% [30.0% - 52.8%] | 78.6% [67.8% - 89.3%] | 76.7% [65.3% - 88.1%] | 74.1% [66.0% - 82.2%] | 75.0% [67.0% - 83.0%] |
| All 6 processes ‡ | N=288 | 41.7% [27.7% - 55.6%] | 39.1% [25.6% - 52.6%] | 16.7% [8.1% - 25.3%] | 15.0% [7.0% - 23.0%] | 33.9% [21.5% - 46.3%] | 33.1% [20.8% - 45.4%] | 26.8% [18.6% - 35.0%] | 29.8% [20.9% - 38.8%] |
| BP measurement, ≥2x in the past 12 months | N=488 | 87.5% [80.3% - 94.7%] | 86.7% [78.8% - 94.6%] | 80.5% [74.6% - 86.4%] | 80.0% [73.8% - 86.2%] | 92.3% [86.4% - 98.2%] | 91.5% [85.0% - 98.0%] | 84.6% [79.0% - 90.3%] | 86.1% [80.6% - 91.6%] |
| Physical activity and diet advice, in the past 12 months | N=494 | 36.3% [25.7% - 46.8%] | 38.9% [27.8% - 49.9%] | 29.5% [22.8% - 36.3%] | 30.8% [23.7% - 37.8%] | 56.3% [45.4% - 67.1%] | 57.3% [46.4% - 68.2%] | 38.6% [31.0% - 46.2%] | 35.4% [27.7% - 43.0%] |

HbA1c: glycated hemoglobin. BP: blood pressure.

Probabilities estimated from logistic regression models (predictive margins). Adjustment: age, sex, living arrangement status, residential location, education level, mandatory health insurance model, subsidies for mandatory health insurance, and diabetes-related complications.

* Only participants who have already heard about HbA1c.

† Among the following: eye examination, foot examination, microalbuminuria screening, blood cholesterol measurement, and influenza immunization.

‡ Among the following: eye examination, foot examination, microalbuminuria screening, blood cholesterol measurement, influenza immunization, and HbA1c measurement.

**Supplementary Table 2:** Estimated outcomes of care, by healthcare use profile

|  |  | **Profile 1 “GP & podiatrist”** | | **Profile 2 “GP only”** | | **Profile 3 “High users”** | | **Profile 4 “Diabetologist first”** | |
| --- | --- | --- | --- | --- | --- | --- | --- | --- | --- |
|  |  | Crude mean [95% CI] | Adjusted mean [95% CI] | Crude mean [95% CI] | Adjusted mean [95% CI] | Crude mean [95% CI] | Adjusted mean [95% CI] | Crude mean [95% CI] | Adjusted mean [95% CI] |
| SF-12, Physical Component Summary score | N=484 | 42.1 [39.8 - 44.4] | 44.0 [41.8 - 46.1] | 45.3 [43.7 - 46.8] | 45.2 [43.7 - 46.6] | 41.7 [39.5 - 44.0] | 43.0 [41.0 - 45.1] | 47.5 [45.9 - 49.1] | 46.0 [44.5 - 47.5] |
| SF-12, Mental Component Summary score | N=483 | 46.9 [44.4 - 49.4] | 45.9 [43.4 - 48.4] | 48.1 [46.4 - 49.7] | 46.7 [45.0 - 48.4] | 43.5 [41.1 - 46.0] | 44.8 [42.4 - 47.3] | 45.4 [43.6 - 47.1] | 46.7 [44.9 - 48.5] |
| ADDQoL score | N=495 | -1.3 [-1.6 - -1.0] | -1.5 [-1.8 - -1.2] | -1.0 [-1.2 - -0.8] | -1.2 [-1.4 - -1.0] | -1.7 [-2.0 - -1.4] | -1.6 [-1.9 - -1.3] | -1.5 [-1.7 - -1.3] | -1.4 [-1.6 - -1.2] |
| PACIC score | N=488 | 2.7 [2.6 - 2.9] | 2.9 [2.7 - 3.1] | 2.4 [2.2 - 2.5] | 2.4 [2.3 - 2.6] | 3.3 [3.1 - 3.4] | 3.3 [3.1 - 3.5] | 2.9 [2.8 - 3.1] | 2.8 [2.7 - 3.0] |
| Diabetes Self-Efficacy Scale score * | N=268 | 7.0 [6.4 - 7.5] | 6.8 [6.2 - 7.4] | 7.6 [7.2 - 8.0] | 7.5 [7.1 - 7.9] | 6.9 [6.4 - 7.4] | 7.1 [6.6 - 7.6] | 7.3 [6.9 - 7.8] | 7.5 [7.0 - 7.9] |
| HbA1c value † | N=240 | 7.0 [6.6 - 7.3] | 7.0 [6.6 - 7.3] | 6.9 [6.6 - 7.2] | 6.9 [6.6 - 7.3] | 7.4 [7.1 - 7.7] | 7.4 [7.1 - 7.7] | 7.3 [7.1 - 7.5] | 7.3 [7.1 - 7.5] |
|  |  |  |  |  |  |  |  |  |  |
|  |  | Crude prob. [95% CI] | Adjusted prob. [95% CI] | Crude prob. [95% CI] | Adjusted prob. [95% CI] | Crude prob. [95% CI] | Adjusted prob. [95% CI] | Crude prob. [95% CI] | Adjusted prob. [95% CI] |
| BP <140/90 mmHg | N=258 | 57.1% [44.2% - 70.1%] | 57.3% [44.5% - 70.2%] | 66.7% [57.2% - 76.1%] | 67.5% [58.1% - 76.8%] | 64.7% [48.6% - 80.8%] | 66.4% [50.4% - 82.4%] | 77.8% [68.2% - 87.4%] | 76.4% [66.0% - 86.7%] |

SF-12: 12-Item Short-Form Health Survey. ADDQoL: Audit of Diabetes-Dependent Quality of Life. PACIC: Patient Assessment of Chronic Illness Care. HbA1c: glycated hemoglobin. BP: blood pressure.

Means (or probabilities) estimated from linear (or logistic) regression models (predictive margins). Adjustment: age, sex, living arrangement status, residential location, education level, mandatory health insurance model, subsidies for mandatory health insurance, and diabetes-related complications.

* Only participants of the 2017 recruitment phase.

† Only participants who have already heard about HbA1c.
